# Effectiveness and efficiency of tele-expertise for improving access to retinopathy screening among 351 neonates in a secondary care center: An observational, controlled before-after study

**DOI:** 10.1371/journal.pone.0206375

**Published:** 2018-10-26

**Authors:** Marie Moitry, Kevin Zarca, Michèle Granier, Marie-Stéphanie Aubelle, Nathanaël Charrier, Brigitte Vacherot, Georges Caputo, Maroua Mimouni, Pierre-Henri Jarreau, Isabelle Durand-Zaleski

**Affiliations:** 1 Laboratoire d’Épidémiologie et de Santé Publique, Strasbourg, France; 2 Service de Santé Publique, Hôpitaux Universitaires de Strasbourg, Strasbourg, France; 3 Assistance Publique-Hôpitaux de Paris, DRCD-URC Eco Ile-de-France (AP-HP), Paris, France; 4 Assistance Publique-Hôpitaux de Paris, service de santé publique, Henri Mondor-Albert- Chenevier, Créteil, France; 5 Service de réanimation néonatale, Hôpitaux Sud Francilien, Evry, France; 6 Service de Médecine et Réanimation Néonatales de Port-Royal, Assistance Publique, Hôpitaux de Paris, Hôpital Cochin, Paris, France; 7 DHU Risques et grossesse, Université Paris Descartes, Paris, France; 8 USRC, Centre Hospitalier René Dubos, Pontoise, France; 9 Service d’ophtalmologie pédiatrique, Fondation Rothschild, Paris, France; 10 Faculté de Médecine, Université Paris-Est & ECEVE UMRS, Créteil, France; Hospital JP Garrahan, ARGENTINA

## Abstract

In France, secondary care hospitals encounter difficulties to adhere to retinopathy of prematurity (ROP) screening guidelines. Our objective was to assess the effectiveness and efficacy of a tele-expertise program for ROP screening in neonatal intensive care units without on-site ophthalmologists. We evaluated the impact of a tele-expertise program funded by the Paris Region Health Authority in a secondary care center general hospital of the Paris Region (CHSF), where there was previously no on-site ophthalmologist. We performed an observational, controlled before-after study, with a university tertiary care center with on-site ophthalmologists (Port-Royal) as the control group. Recruitment and data collection for both periods took place from 1 January 2012 to 31 December 31 2012, and from 1 January 2014 to 31 March 2015. The primary endpoint was the percentage of compliance with screening guidelines, secondary endpoints included pain scores and costs. Over the two periods, at total of 351 infants were recruited in the CHSF. Implementation of the tele-expertise resulted in an absolute +57.3% increase in the proportion of examinations realized in accordance with guidelines (3.8% during the "before" period and 61.1% during the "after" period, p<0.001). As compared with the control group, the proportion of infants appropriately screened improved (57.5% versus 43.1%, p = 0.002); median pain score on the acute pain rating scale for neonates during examination was significantly higher (median score 5.5/10, range [2.5–5.7] versus 2.0/10, range [1.0–3.1], p = 0.002). Screening rates in the control group remained unchanged. The average cost per examination increased from €337 in the "before" period to €353 in the "after period" in the tele-expertise group. The implementation of tele-expertise for ROP screening in the CHSF medical center resulted in a major improvement of access to care with a small cost increase. The issue of pain control during examination with tele-expertise should be further addressed.

## Introduction

Retinopathy of prematurity (ROP) is a development disorder of the retina vasculature that affects approximatively 184,700 infants worldwide, with more than 50,000 progressing to potentially visual-impaired diseases [1]. In high-income countries, it affects 15.1% of surviving infants born before a Gestational Age (GA) of 32 weeks and is a leading cause of childhood blindness. ROP progression to visual impairments can be largely avoided with treatments that have proved their effectiveness [2,3] provided that screening is performed in accordance with the recommended guidelines: at post menstrual age of 31 weeks for infants born with GA of less than 28 weeks, at post menstrual age of 32 weeks for infants born with GA of 28 weeks, and at 4 weeks of chronological age for infants born after GA of 28 weeks (American Academy of Pediatrics, [4]). In France, as hospital-based ophthalmologists are increasingly rare with less than 800 in Metropolitan France in 2016 [5], tertiary as well as secondary care hospitals encounter difficulties to adhere to screening guidelines. These hospitals suffer from recurrent delays in screening and in treatment of retinal lesions. To address this issue of delayed access to care, a pilot tele-expertise project was implemented by the regional health authority in a secondary care center of the Paris region [6]. The screening consisted in a fundus examination performed with a digital camera and remote interpretation of images by an experimented ophthalmologist. Considering that all pediatricians should be able to handle the camera for providing pictures analyzed by an expert and that tele-expertise for ROP screening has recently shown good results in both effectiveness and cost-effectiveness [7,8], we hypothesized that it would substantially improve ROP screening in the CHSF center with some increase in cost due to the current high costs of its required equipment. Our objective was to assess the effectiveness and efficiency of the implementation of tele-expertise program for ROP screening in neonatal intensive care units without on-site ophthalmologists.

## Material and methods

### Participants

Infants born before GA of 33 weeks and/or with a birth weight inferior to 1500 grams hospitalized in neonatal intensive care units (NICU) and without cerebral malformations were eligible. Infants deceased during their hospitalization were not included.

### Study design and settings

The implementation of a telemedicine program was decided by the regional health authority in a general hospital located in the South of the Paris Region (CHSF, Centre Hospitalier Sud Francilien, 5,163 deliveries in 2015 [9]), where there was no on-site ophthalmologist. We performed an observational, controlled before-after study with a university tertiary care center, located in the center of Paris (Port-Royal, 5,036 deliveries in 2015 [9]), and staffed with dedicated pediatric ophthalmologists who performed on site examinations as the control group. Recruitment in the "before" period took place from 1 January 2012 to 31 December 2012, and in the "after" period from 1 January 2014 to 31 March 2015.

### Standard procedure

Ophthalmologists performed a funduscopic examination using a binocular indirect ophthalmoscopy technique. In the CHSF center, as there was no on-site ophthalmologist, premature infants were either transported to a tertiary care center with specialized pediatric ophthalmologists, before their discharge from the hospital–providing a consultation was available at that time–or after discharge. In the control university tertiary care hospital, the on-site pediatric ophthalmologist visited the ICU department for eye examination.

### Tele-expertise procedure

The first step of the examination was to perform a topical anesthesia with one drop of oxibuprocaine chlorohydrate instilled in each eye. Pupils were dilated 45 minutes before the examination with a combination of one instillation of 2.5% phenylephrine and 3 instillations of 0.5% tropicamide eye drops given 15 minutes apart. A few minutes before the examination, oral saccharose was given by sucking with a pacifier. A drop of local vasoconstrictor (phenylephrine) and an eyelid speculum (disposable or sterilized, suitable to child's size) were applied, and then a contact gel so that the camera could be placed over the cornea. After the pictures were taken with the Retcam, the camera was cleaned with an aqueous solution of sodium hypochlorite and sodium chlorite then rinsed with sterile water. The speculum was removed and cardiovascular and respiratory functions were monitored for at least 30 minutes for a hospitalized infant and one hour for an infant coming for an external visit. Once the pictures were uploaded to a secure server, they were reviewed and interpreted on a computer by a specialized pediatric ophthalmologist of the ophthalmologic department of the Rothschild foundation. No other resources were required.

### Outcomes and data sources

We used the MAST model (Model of Assessment of Telemedicine Applications) [10] in order to perform a multidimensional assessment. MAST is a framework for assessing the value of telemedicine that is based on the core health technology assessment model. We collected results of clinical effectiveness, patient perspective, economic aspects and organizational aspects.

### Effectiveness

Data were collected in both centers (CHSF and Port-Royal) during both periods (before and after). The following variables were recorded: sex, birth date, GA, birth weight, height and head circumference, dates of entry and discharge from NICU, date of eye examination, procedure (tele-expertise or usual care) and destination at hospital discharge (transfer to another center, return home, death). The primary outcome was the proportion of infants having a ROP screening within the guidelines of the American Academy of Pediatrics [4]. We considered that examination satisfied the primary outcome if it occurred at the theoretical recommended date +/- 6 days. The secondary endpoint was deviation from guidelines computed as the interval between recommended and effective date of examination.

### Patient perspective

Independently of the main study, a small sample of infants was recruited during the "after" period in the CHSF (tele-expertise) and Port-Royal (on-site examination) hospitals to compare pain scores between the two techniques. The following variables were recorded using a specific case report form: GA at birth and at examination date, weight at examination date, drug or non-drug treatments administrated, heart rate, saturation oxygen levels and pain scores before, during and after examination. We assessed pain scores during eye examination based on the validated APN pain scale (Acute Pain rating scale for term and preterm Neonates) [11]. This scale evaluates three items: facial expression, limb movements, and vocal expression with ratings per item ranging from 0 to 4, 0 to 3 and 0 to 3, respectively.

### Cost analysis

Costs before and after the implementation of tele expertise were estimated for the Centre Hospitalier Sud Francilien. The cost analysis was conducted from the hospital perspective using tariffs as a proxy for the costs when production costs were not accessible. Unit costs are presented in Table 1. All costs are in 2017 euros (US$1 = €0.754). For usual care (before period in the CHSF), as there was no on-site ophthalmologist, the operating cost of one request was calculated by adding the cost of transportation to the tariff of the examination.

**Table 1:** Unit costs used for the economic evaluation.

**Usual care**	**Unit cost**	**Source**
Tariff (per unit)	37 €	Social health insurance schedule
Transportation (round-trip, per unit)	300 €	Social health insurance schedule
**Tele-expertise**	**Unit cost**	**Source**
Staff time (per unit)	143 €	2010 hourly salary of the CHSF
Retcam	114 338 €	Financial department of the CHSF
Software	40 945 €	Financial department of the CHSF
Camera maintenance	7 774 €	Financial department of the CHSF
Software maintenance	2 414 €	Financial department of the CHSF
Software subscription	4 306 €	Financial department of the CHSF

Investment costs associated with tele-expertise comprised the acquisition of the camera and the software. Operating costs included costs of maintenance of the camera and software subscription. Human resources costs were obtained with a micro-costing top-down approach, based upon time needed for one examination in the requesting (CHSF hospital) and the specialist institution (Rothschild Foundation). Overall cost of a fundus examination was calculated with respect to the number of requests per year, on the basis of a 5-year depreciation period and a 4% discount rate for the tele-expertise equipment.

### Sensitivity analysis

To assess the cost drivers of eye examination using the Retcam, we performed several scenario analyses by varying the following parameters: purchase price of the Retcam (from -50% to +10%), completion time for one request (from -20% to +20%), qualification of the two health care professionals taking the pictures with the camera (whether it was one physician and one nurse or two nurses; whether the physician was a fellow or a professor), depreciation period (from 2 to 10 years) and discount rate (from 0% to 5%). We plotted results from scenario analyses on a tornado diagram.

### Trial registration and ethics approval

Clinicaltrials.gov: NCT02157727

The ethics approval was given by the “Comité Consultatif sur le Traitement de l’Information en matière de Recherche dans le domaine de la Santé” (CCTIRS), number 15.162. It approved the lack of parent or guardian consent in the decision, as every effort has been made to provide information to parents.

Data were fully unidentified prior access to authors.

### Statistical methods

Data were described as numbers and percentages, means and standard deviations or medians and ranges. Comparisons were performed using Chi-squared test, Student or Kruskall and Wallis tests, as appropriate. We performed intention-to-treat analyses. For the primary outcome, a logistic regression model was used to assess effectiveness of the intervention adjusted on sex and (GA) at birth. All tests were two-tailed and significance level was set at 0.05. Analyses were carried out using SAS version 9.3 (SAS Institute, Cary, NC, USA).

## Results

### Overview

In the CHSF hospital, 158 infants were recruited during the "before" period. During the "after" period, among the 193 infants included, 149 were examined with the Retcam (77.2%). In the hospital center of Port-Royal, 217 and 269 infants were recruited respectively during the "before" and "after" periods.

No significant differences regarding distributions by sex, GA and birth weight at baseline were observed between the "before" and "after" periods in the CHSF hospital (Table 2). In the Port-Royal hospital, distribution of infants according to gestational age (GA) at birth significantly differed between the before and the "after" period (Table 3). The proportion of infants with a birth weight lower than 1500g decreased between the two periods (98.6% versus 82.9%, p<0.001) despite an increase in absolute number (223 versus 214).

**Table 2:** Infants characteristics and outcomes for effectiveness—CHSF medical center, before and after periods.

	Before	After	
	N = 158	N = 193	
	N (%)	N (%)	p
Sex (Girl)	82 (51.9%)	85 (44.0%)	0.14
Gestational Age			
< 27 weeks	20 (12.7%)	29 (15.0%)	0.81
27–29 weeks	64 (40.5%)	75 (38.9%)	0.81
30 weeks and more	74 (46.8%)	89 (46.1%)	0.81
Birth Weight <1500 g	126 (79.7%)	153 (79.3%)	0.91
Compliance with guidelines	6 (3.8%)	118 (61.1%)	<0.001*
	**Mean (SD)**	**Mean (SD)**	
Deviation from guidelines (days)	64.3 (42.0)	7.9 (19.3)	<0.001*

SD: Standard Deviation

*Significant

**Table 3:** Infants characteristics and outcomes for effectiveness—Port-Royal medical center, before and after periods.

	Before	After	
	N = 217	N = 269	
	N (%)	N (%)	p
Sex (Girl)	112 (51.6%)	140 (52.0%)	0.92
Gestational Age			
< 27 weeks	42 (19.4%)	45 (16.7%)	0.007*
27–29 weeks	98 (45.2%)	91 (33.8%)	0.007*
30 weeks and more	77 (35.5%)	133 (49.4%)	0.007*
Birth Weight <1500 g	214 (98.6%)	223 (82.9%)	<0.001*
Compliance with guidelines	93 (42.9%)	120 (44.6%)	0.70
	**Mean (SD)**	**Mean (SD)**	
Deviation from guidelines (days)	15.1 (34)	11.9 (24.7)	0.30

SD: Standard Deviation

*Significant

### Work flow

**Fig 1:**
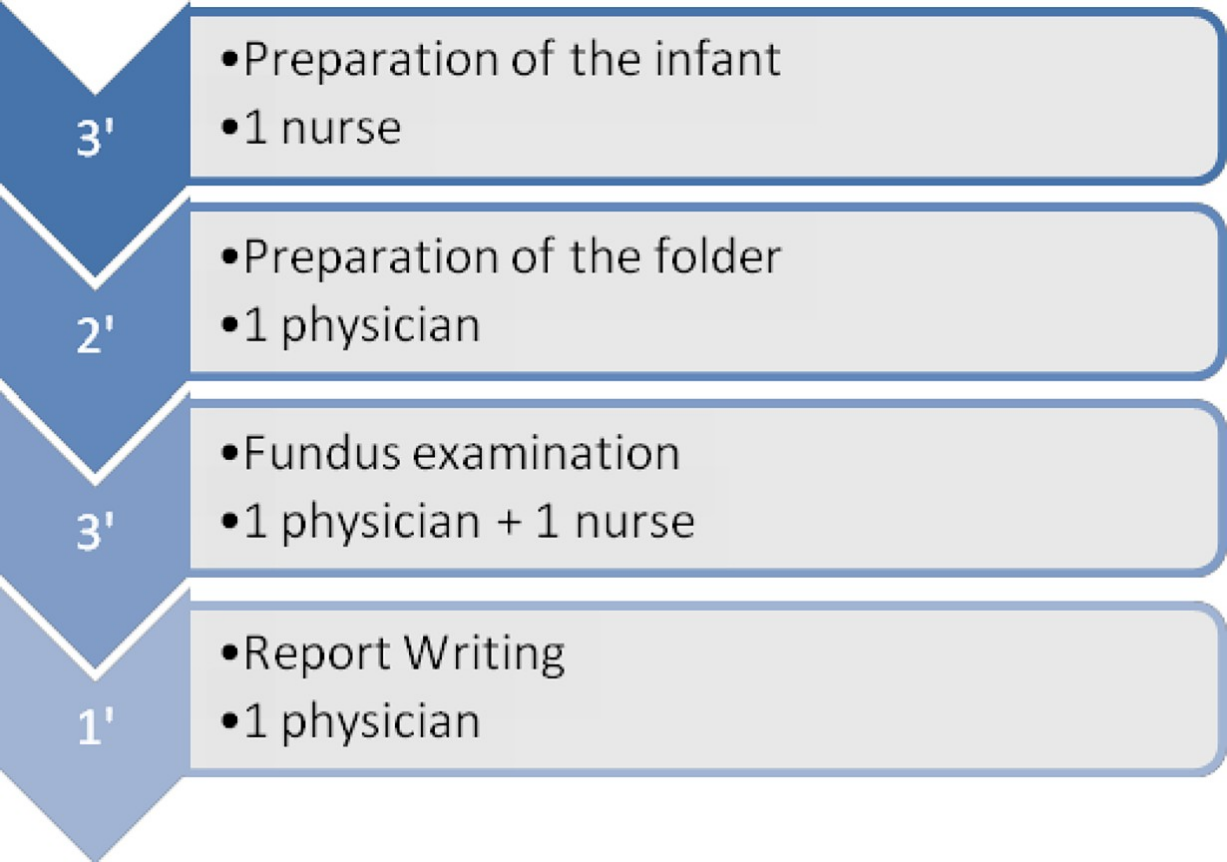
Workflow, standard procedure (minutes).

**Fig 2:**
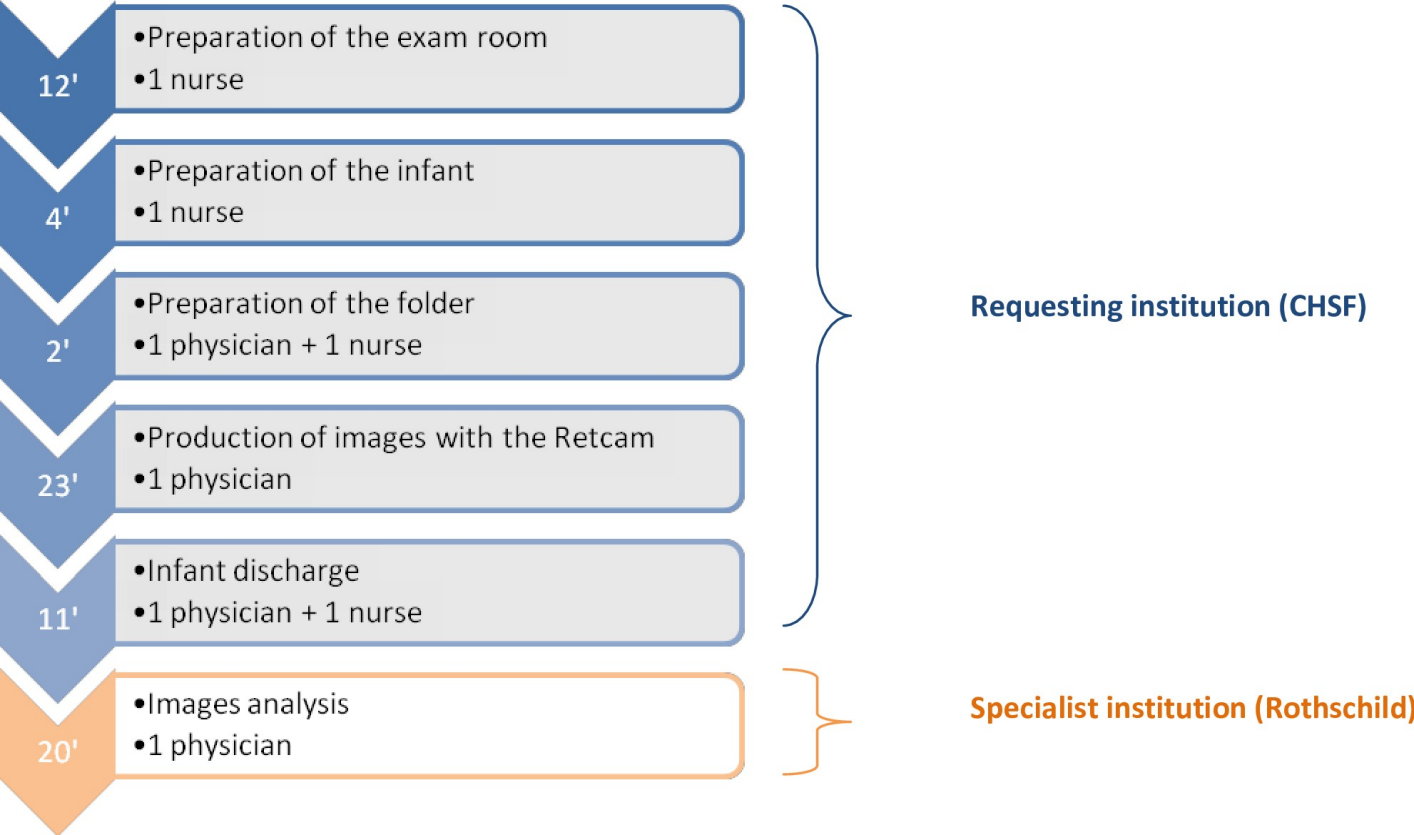
Workflow, tele-expertise (minutes).

The workflows for one examination with and without tele-expertise are detailed in Figs 1 and 2. For the standard procedure, depending on the on-course step, it mobilized either one physician, one nurse, or both and lasted an average of 9 minutes (bottom-up approach). Because of losses of ttime experimented between two examinations (i.e. solicitation of the physician or the nurse for other tasks) or examinations that sometimes lasted longer than expected, the number of examinations actually performed during a 120-minutes timeframe (top-down approach) was estimated at 8. With tele-expertise, an average of 72 minutes was necessary for eye examination performance and analysis, with about 23 minutes for taking the pictures. The ophthalmologist of the specialist institution (Rothschild) indicated that during a 240-minutes timeframe, an average of 8 patients were analyzed, corresponding to a 30 minutes examination.

### Effectiveness

Implementation of the tele-expertise resulted in an absolute +57.3% increase in the proportion of fundus examinations realized in accordance with the guidelines (3.8% in the "before" period versus 61.1% in the "after" period, p<0.001) as well as a significant decrease in the deviation from guidelines regarding the time between birth and examination (64.3 days versus 7.9 days, p<0.001) (Table 2) Multivariate analyses confirmed that implementation of tele-expertise significantly increased the probability for infants to be appropriately screened (OR = 52.2 CI95 [21.0–129.4], p<0.001).

Between the two periods in the control group (Port-Royal), we found no significant difference relative to the primary outcome: 42.9% (N = 93) of infants were screened in accordance with guidelines during the "before" period as compared with 44.6% (N = 120) during the "after" period. The percentage of appropriate screening in the control hospital was significantly lower than the 61.1% observed in the CHSF medical center (p = 0.002, Table 4). Multivariate analysis confirmed this finding (OR = 1.77 CI95 [1.19–2.63]).

**Table 4:** Infants characteristics and outcomes for effectiveness—CHSF and Port-Royal medical centers, after period.

	Port-Royal	CHSF	
	N = 269	N = 193	
	N (%)	N (%)	p
Sex (Girl)	140 (52.0%)	85 (44.0%)	0.09
Gestational Age			
< 27 weeks	45 (16.7%)	29 (15.0%)	0.53
27–29 weeks	91 (33.8%)	75 (38.9%)	0.53
30 weeks and more	133 (49.4%)	89 (46.1%)	0.53
Birth Weight <1500 g	223 (82.9%)	153 (79.3%)	0.32
Compliance with guidelines	120 (44.6%)	118 (61.1%)	<0.001*
	**Mean (SD)**	**Mean (SD)**	
Deviation from guidelines (days)	11.9 (24.7)	7.9 (19.3)	0.09

SD: Standard Deviation

*Significant

### Patient perspective

Data on pain during examination were collected for 56 infants (13 in Port-Royal and 43 in the CHSF center). For 2 infants, data were missing. Characteristics of infants according to the center are detailed in Table 5. No significant differences regarding distributions by GA at birth, or birth and weight at examination date observed between the Port-Royal and the CHSF medical centers (Table 2). Drug and non-drug treatments were almost systematically administrated for eye examination with the RETCAM. Median pain score evaluated during examination was significantly higher among infants examined with tele-expertise as compared with infants receiving usual eye examination (5.5/10, range [2.5–5.7] versus 2.0/10, range [1.0–3.1], p = 0.002). Heart rates were also increased when the RETCAM was used (190. 0 range [184.0–200.0] versus 169.5 range [15.0–181.5], p = 0.0002).

**Table 5:** Infants characteristics and outcomes for pain evaluation—CHSF and Port-Royal medical centers, after period.

	Port-Royal	CHSF	
	N = 13	N = 41	p
	Median [q1—q3]	Median [q1—q3]	
Gestational age at birth (weeks)	26.2 [24.6–28.6]	27.5 [26.6–29.0]	0.08
Gestational age at examination date (weeks)	35.4 [32.3–37.9]	35.5 [34.3–37.9]	0.62
Weight at examination age (grams)	2050.0 [1570.0–2396.0]	2070.0 [1662.5–2330.0]	0.99
Saturation (%)			
During examination	93.5 [78.5–99.5]	97.0 [95.0–98.0]	0.11
Before examination	95.5 [89.0–99.0]	98.0 [95.0–100.0]	0.18
After examination	96.0 [82.5–99.5]	98.0 [97.0–100.0]	0.07
Heart rate (beats per minute)			
During examination	169.5 [15.0–181.5]	190.0 [184.0–200.0]	0.0002*
Before examination	168.0 [154.0–175.5]	155.0 [145.0–168.0]	0.13
After examination	169.5 [159.5–182.0]	170.0 [160.0–177.0]	0.74
Drug and non-drug therapies	**N (%)**	**N (%)**	** **
Anesthesia	41 (100.0%)	1 (7.7%)	<0.0001*
Nipple	40 (97.6%)	2 (15.38%)	<0.0001*
Body wraping	41 (100%)	11 (84.6%)	0.054
Pain scores (/10)	Median [q1—q3]	Median [q1—q3]	
During examination	2 [1.0–3.1]	5.5 [2.5–5.7]	0.002*
Before examination	0.0 [0.0–0.9]	0.0 [0.0–0.1]	0.33
After examination	0.0 [0.0–0.4]	0.0 [0.0–0.4]	0.06

q: quartile

*Significant

### Cost analysis

**Fig 3:**
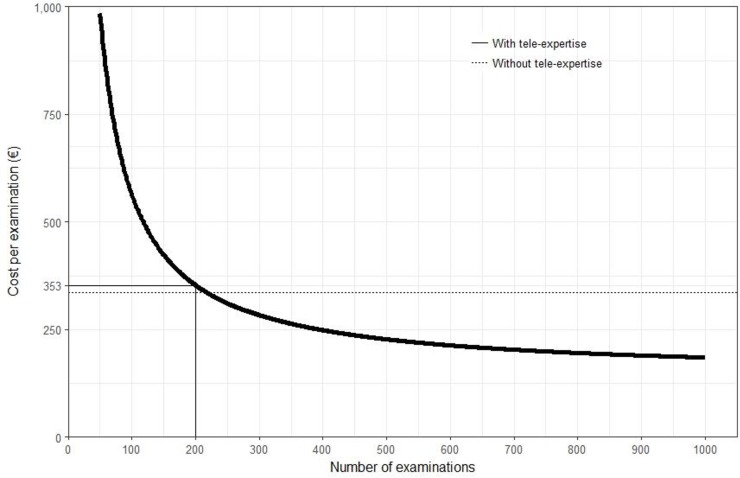
Overall cost of tele-expertise examination as a function of the number of requests (base case-cost curve)–CHSF medical center.

**Fig 4:**
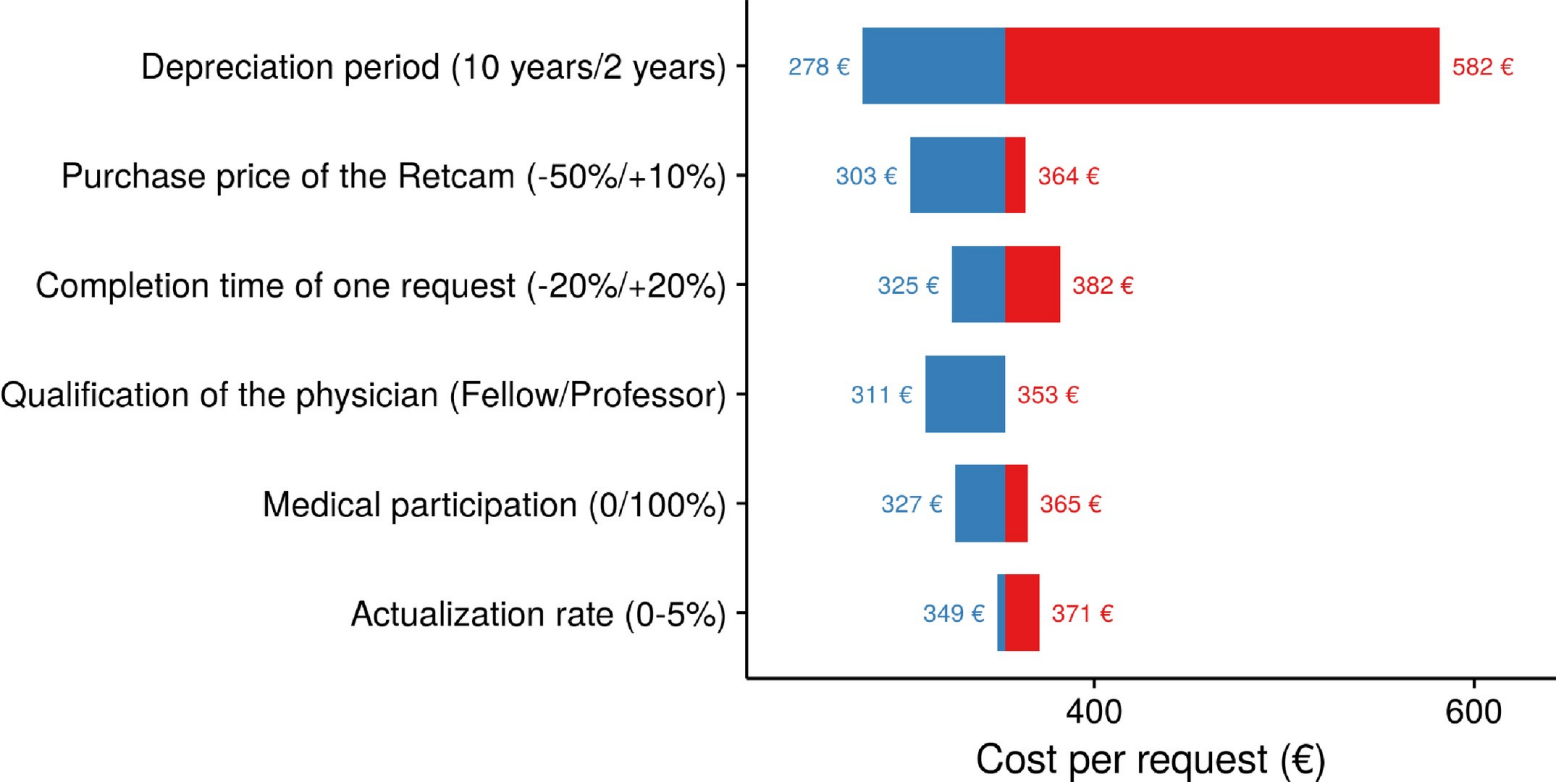
Sensitivity analysis–baseline €353 tele-expertise group.

Overall cost of one eye examination using the standard procedure (transfer to the specialized center and examination by a specialist) was estimated to be 337€. On the basis of 200 examinations per year, it reached €353 with tele-expertise (base-case curve, Fig 3). Results of scenario analyses are presented on the tornado diagram in Fig 4. It appeared that depreciation period had the greatest impact on the overall cost of one examination, which ranged from €582 for a 2-years depreciation period to €278 for a 10-years period.

## Discussion

The implementation of a tele-expertise procedure in a hospital center with a large maternity ward and without an on-site ophthalmologist was effective with an absolute increase of +57.3% of infants screened in accordance with guidelines for the diagnosis of retinopathy of prematurity. This major improvement was confirmed when analysis was adjusted on age at birth and sex. The overall cost, taking account investment and operating costs were almost the same (€337 for the transfer and examination of newborns in a specialized center vs €353 for the onsite examination and tele-expertise).

All around the world, several other telemedicine experiments have been described in countries where either the long distances or the lack of trained specialists is a severe limitation to compliance with ROP screening guidelines [12,13]. These are not necessarily developing countries but for example USA [14], Canada [15], Australia [16], Chile [17] and India [18]. Our results are consistent with previous findings and provide new arguments in favour of tele-medicine programs in understaffed hospitals.

Several methodological aspects require to be addressed. First, the choice of the outcome for effectiveness can be discussed. In the end, the goal of screening is to identify infants that need to be treated for ROP. Respect of screening timetable is a surrogate endpoint that does not directly predict the effect of the Intervention on the reduction of ROP incidence. However, in this study, we addressed the value of tele-expertise from a public health perspective. The objective was not to assess effectiveness of such a program to detect and treat ROP, which has been largely reviewed [13,19–21], but whether its implementation enabled an improvement in access to care in medical centers with limited medical resources. We therefore did not collect data on examination results or treatments.

One of the limits of our study was that costs for an examination were not prospectively collected for every infant, and individual costs of procedures could therefore not be estimated. In particular, for the "before" period in the CHSF medical center, we had no information on medical transportation after patient discharge, and the cost analysis was performed assuming that the performance of one examination systematically implied medical transportation.

Infants that underwent RETCAM examination faced a higher pain level during examination, with a median score reaching 5.5/10 versus 2.0/10 with a standard examination. This can be explained by a longer examination time (Fig 2), by a greater mobilization of the child (local anesthesia, pupillary dilation, placing of a lid speculum, application of the camera on the eye, etc.) and by professionals' lack of practice at the time of the study. A few studies compared pain of infants during examination with and without tele-expertise and found differing results, suggesting that there is still room for improvement of pain control [22–25].

## Conclusions

The implementation of tele-expertise for ROP screening in the CHSF medical center resulted in a major improvement of access to care with only a small cost increase. In the future, costs using tele-expertise could be reduced by negotiating the purchase price of equipment or implementing specific training for physicians or non physicians [26,27]. Patient outcomes, especially regarding pain control, are yet to be further assessed and improved [21].

## Abbreviations

ROP: Retinopathy of prematurity
NICU: Neonatal intensive care units
CHSF: Centre Hospitalier Sud-Francilien
PMA: Post Menstrual Age
GA: Gestational age
MAST: Model for Assessment of Telemedicine Applications
APN: Acute Pain rating scale for term and preterm Neonates
